# GPCR-mediated effects of fatty acids and bile acids on glucose homeostasis

**DOI:** 10.3389/fendo.2023.1206063

**Published:** 2023-07-07

**Authors:** Antwi-Boasiako Oteng, Liu Liu

**Affiliations:** Molecular Signaling Section, Laboratory of Bioorganic Chemistry, National Institute of Diabetes and Digestive and Kidney Diseases, National Institutes of Health (NIH), Bethesda, MD, United States

**Keywords:** bile acids, enteroendocrine cells, FFAR1, FFAR2, FFAR3, FFAR4, fatty acids, glucagon

## Abstract

Fatty acids and glucose are key biomolecules that share several commonalities including serving as energy substrates and as signaling molecules. Fatty acids can be synthesized endogenously from intermediates of glucose catabolism via de-novo lipogenesis. Bile acids are synthesized endogenously in the liver from the biologically important lipid molecule, cholesterol. Evidence abounds that fatty acids and bile acids play direct and indirect roles in systemic glucose homeostasis. The tight control of plasma glucose levels during postprandial and fasted states is principally mediated by two pancreatic hormones, insulin and glucagon. Here, we summarize experimental studies on the endocrine effects of fatty acids and bile acids, with emphasis on their ability to regulate the release of key hormones that regulate glucose metabolism. We categorize the heterogenous family of fatty acids into short chain fatty acids (SCFAs), unsaturated, and saturated fatty acids, and highlight that along with bile acids, these biomolecules regulate glucose homeostasis by serving as endogenous ligands for specific G-protein coupled receptors (GPCRs). Activation of these GPCRs affects the release of incretin hormones by enteroendocrine cells and/or the secretion of insulin, glucagon, and somatostatin by pancreatic islets, all of which regulate systemic glucose homeostasis. We deduce that signaling induced by fatty acids and bile acids is necessary to maintain euglycemia to prevent metabolic diseases such as type-2 diabetes and related metabolic disorders.

## 1 Introduction

Dysregulation of glucose metabolism leads to an array of metabolic disorders, including obesity and type-2 diabetes. These two chronic disorders rank amongst the top causes of morbidity and mortality worldwide (1–3). Several biological processes influence glucose metabolism, not least amongst them is lipid metabolism. Glucose homeostasis is intricately linked with lipid metabolism because glucose can serve as substrate for endogenous synthesis of fatty acids by de-novo lipogenesis. Moreover, fatty acids can compensate for glucose deficiency by replacing glucose as the source of oxidative fuel in several metabolic tissues.

This review focusses on mechanisms by which exocrine and endocrine molecules that are secreted in response to lipid ingestion activate cell surface receptors that regulate systemic glucose homeostasis. One such exocrine factor is bile which is released into the intestine to aid emulsification, hydrolysis and uptake of ingested lipids. Bile originates from hepatocytes and is composed of bile acids/salts, bilirubin phospholipid, cholesterol, amino acids, steroids, enzymes, porphyrins, vitamins, and heavy metals (4). The bile acid component of bile, and the fatty acids from intestinal fat hydrolysis possess endocrine properties, and thus serve as ligand activators of specific GPCRs on enteroendocrine cells. This leads to release of gut-specific hormones such as glucagon-like peptide 1 (GLP-1) and glucose-dependent insulinotropic polypeptide (GIP), that are crucial to systemic glucose homeostasis by activating their respective receptors on pancreatic beta cells to promote insulin secretion. Accordingly, several pharmacological agents that mimic the beneficial effects of bile and fatty acids against metabolic diseases via GPCRs are under clinical investigation to treat obesity-associated diseases such as type-2 diabetes (5–7).

It is worth mentioning that, under pathophysiological conditions such as obesity and type-2 diabetes, the circulating levels of fatty acids and bile acids are altered. Whereas fatty acid levels are elevated in obesity and type-2 diabetes (8–10), higher levels of circulating bile acids have been reported in persons who have undergone bariatric surgery to correct obesity (11, 12). However, and as discussed below, the individual fatty and bile acids differ in their physiological functions. Therefore, their physiological benefits or detrimental impact on metabolic diseases depend on the extent of altered levels of specific bile or fatty acids under different pathophysiological conditions. There are different sub-classifications of bile acids based on structural modifications that occur as bile acids travel along the enterohepatic path. Similarly, there is heterogeneity among dietary and endogenously synthesized fatty acids based on length of carbon chain, degree of saturation, or cis-trans bond configuration. These structural differences influence the potency of specific bile and fatty acids to serve as ligands for specific GPCRs that affect glucose homeostasis.

This review highlights the heterogeneity amongst bile and fatty acids, and addresses how GPCR-mediated signaling by these biomolecules regulate systemic glucose homeostasis. Clearly, a deeper understanding of the regulation of systemic glucose metabolism via lipid-derived signaling molecules can unravel novel therapeutic targets, and enhance approaches to treat diseases characterized by aberrant glucose and lipid metabolism.

### 1.1 Fatty acids

Fatty acids are a class of lipids characterized by a hydrophobic hydrocarbon chain and a terminal carboxylic acid functional group. Fatty acids can be categorized based on hydrocarbon chain length, number and position of double bonds (unsaturation), and the presence of cis or trans double bonds. The length of the hydrocarbon chain defines fatty acids as either short, medium, or long chain. Typically, short chain fatty acids (SCFAs) have up to 4 carbon atoms in the hydrocarbon backbone, 6 to 12 carbon fatty acids are medium chain, whereas 13 to 21 carbon fatty acids are considered long chain fatty acids (LCFAs). The degree of saturation defines fatty acids as either saturated with no double bonds, or unsaturated with at least one double bond. Unsaturated fatty acids with a single double bond are mono-unsaturated whereas those with two or more double bonds are polyunsaturated fatty acids (PUFAs). In a cis-unsaturated fatty acid, all double bonds are in cis-configuration, whereas the presence of at least one trans double bond defines a trans-unsaturated fatty acid (13). Additionally, PUFAs occur naturally as omega-3 or omega-6 depending on the position of the last double bond from the terminal carbon.

Each day, the human body encounters and processes dietary fat of varying composition and quantities, due to the heterogeneity of our diet. Following ingestion and digestion of fat-rich foods, fatty acids are absorbed via the epithelial enterocytes that line the small intestine, re-esterified into triglycerides, and packaged as chylomicrons for onward secretion into circulation via the lymph. Inside the lumen of blood vessels, the triglyceride component of chylomicrons is hydrolyzed by lipoprotein lipase, to release free fatty acids, which are then taken up by various tissues as energy-providing substrates, or for storage as fat. Moreover, lipolysis occurring in adipose tissue, especially after prolonged fast, contributes to circulating levels of fatty acids. These fatty acids bind to albumin to enhance their hydrophilicity and circulation to specific tissues. The third source of circulating fatty acids are very-low density lipoprotein (VLDL) secreted from the liver. Gut epithelial cells and tissues that utilize fatty acids as a fuel source or for storage encounter and respond to different fatty acids via specific receptors, leading to signaling outcomes that affect glucose homeostasis (14, 15).

### 1.2 Bile acids

Bile acids are biological detergents which are synthesized in the liver from cholesterol, and stored in the gall bladder until released into the gut upon food intake to aid the emulsification, digestion and absorption of dietary cholesterol, fat, and other lipophilic nutrients (16, 17). Upon synthesis in the liver, primary bile acids such as cholic acid, chenodeoxycholic acid, hyocholic acid, and murine-specific muricholic acid can be conjugated to either taurine or glycine. In the small and large intestine, gut microbial enzymes deconjugate and metabolize the primary bile acids into secondary bile acids such as deoxycholic acid, lithocholic acid, and ursodeoxycholic acid (17). Due to their important role in lipid metabolism, bile acids have an indirect effect on glucose homeostasis (16, 18). Additionally, several studies have reported a more direct effect of specific bile acids on glucose metabolism due to their ability to activate specific GPCRs, such as G-protein bile acid receptor 1 (GPBAR1) (alternative name: Takeda G-protein receptor 5, TGR5) (19, 20).

### 1.3 GPCRs responsive to fatty acids and bile acids

GPCRs are transmembrane receptors which, upon binding of extracellular ligands, couple to heterotrimeric guanine-nucleotide binding proteins (G-proteins) (21). The heterotrimeric G-protein contains beta and gamma subunits, but it is the alpha subunit that primarily defines signaling outcomes and determines the subclassification of G-proteins. GPCRs are very important drug targets for numerous diseases including cancer, and metabolic and neurological disorders, evidenced by the fact that over 30% of all FDA-approved drugs target GPCRs (22). In the context of obesity and diabetes, a recently approved drug, tirzepatide, is highly efficacious in reducing body weight and decreasing HbA1c levels (23, 24). Tirzepatide is a dual agonist for two GPCRs, the GLP-1 and GIP receptors which shows superior ability to lower blood glucose and body weight compared to GLP-1R mono-agonists such as semaglutide and liraglutide (24, 25). Besides these two receptors, other GPCRs have been identified as promising targets for metabolic diseases, including the SCFA receptors, free fatty acid receptor 2 (FFAR2; alternate name GPR43) and free fatty acid receptor 3 (FFAR3; alternate name GPR41), the LCFA receptors, free fatty acid receptor 1 (FFAR1; alternate name GPR40) and free fatty acid receptor 4 (FFAR4; alternate name GPR120) and the bile acid receptor, GPBAR1 (26).

**Figure 1:**
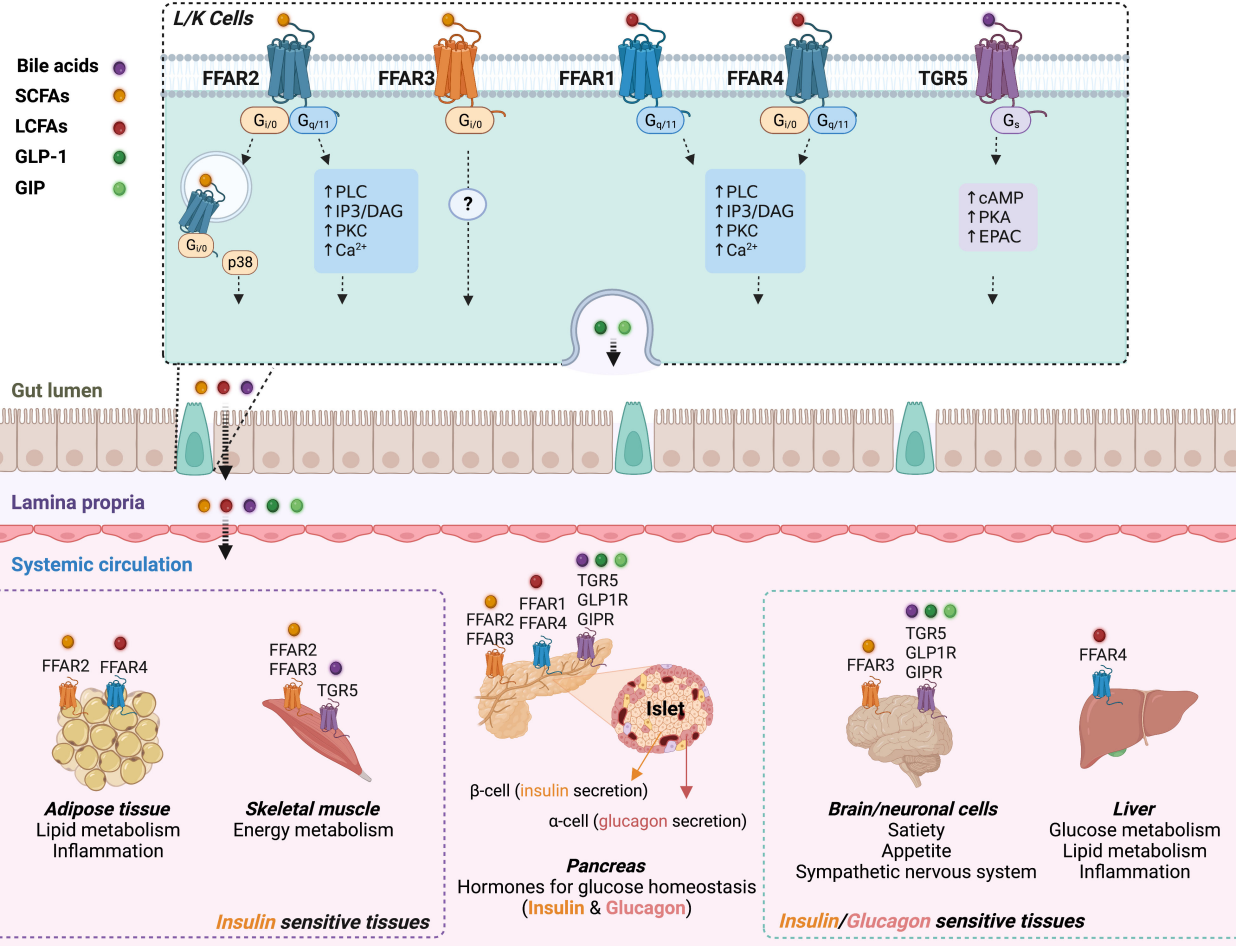
Schematic overview of the interaction between fatty acids or bile acids and their respective GPCRs in the gut, pancreas, adipose tissue, skeletal muscle, liver, and brain. Top panel: Bile acids (purple dot), and nutrient-derived SCFAs (yellow dot) and LCFAs (red dot) interact with their respective GPCRs expressed by enteroendocrine cells, including GLP-1-secreting L cells and GIP-secreting K cells. Fatty acids and bile acids promote release of GLP-1 (deep green dot) and GIP (light green dot) into systemic circulation. Bottom center: GPCR-specific signaling effects of the incretins, circulating fatty acids and bile acids, on insulin and glucagon secretion from pancreatic islets. GLP-1 and GIP stimulate insulin secretion via Gs-signaling after activation of GLP-1R and GIPR in beta cells. Circulating LCFAs and bile acids enhance insulin and/or glucagon secretion via Gq and Gs signaling, respectively. Activation of FFAR2 or FFAR3 by SCFAs regulates insulin secretion via Gq/Gi-coupling. Insulin and glucagon modulate systemic glucose homeostasis through their effects on insulin-responsive tissues (bottom left: adipose tissue and skeletal muscle; bottom right: brain and liver) and glucagon response tissues (bottom right: brain and liver). Bottom left: adipose tissue also expresses FFAR2 (inhibits lipolysis and stimulates adipokine production) and FFAR4 (anti-inflammatory). SCFAs also modulate energy metabolism in skeletal muscle through FFAR2/FFAR3 (see review (30)). Bottom right: GLP-1/GIP and fatty acids/bile acids mediate gut-brain communication through corresponding GPCRs to regulate satiety and appetite, and the sympathetic nervous system (see reviews (31, 32)). FFAR4 is highly expressed in Kupffer cells of the liver, and may affect systemic glucose homeostasis via its potent anti-inflammatory effects (33).

FFAR2 and FFAR3 share ~43% amino acid sequence identity and poor ligand selectivity (27). Both receptors are highly expressed in pancreas and immune cells, in both mice and humans (28), whereas FFAR2 shows preferential expression in adipose tissue, intestine, especially in the ileum and colon (28, 29). FFAR2 shows higher potency for the shorter carbon chain fatty acids, acetate and propionate, while FFAR3 preferentially binds butyrate and propionate. Upon ligand binding, FFAR2 couples to both inhibitory Gαi/o and stimulatory Gαq/11 G proteins, while activation of FFAR3 exclusively induces Gαi/o signaling (**Figure 1**). At the plasma membrane, activated Gαq/11 stimulates phospholipase C (PLC) resulting in the production of diacylglycerol (DAG) and inositol 1,4,5-trisphosphate (IP3), leading to the activation of protein kinase C (PKC) and elevated intracellular calcium (Ca^2+^) levels, respectively. In contrast, activated Gαi/o inhibits adenylyl cyclase, causing a decrease in cyclic AMP (cAMP) production and reduced protein kinase A (PKA) activity (34).

FFAR4 and FFAR1 are activated by medium- and long-chain fatty acids. FFAR4 is well expressed by cells of the gastrointestinal tract, pancreatic islets, and adipose tissue of mice and humans (35) whereas FFAR1 is predominantly expressed in pancreatic islets and brain neuronal cells of mice and humans (36–38) and also reportedly expressed in mouse enteroendocrine cells (39). Ligand-activated FFAR1 and FFAR4 couple to Gαq/11, leading to the activation of the PLC-DAG-IP3- Ca^2+^ signaling network, that can promote the release of hormones from intracellular vesicles of endocrine cells (**Figure 1**).

The bile acid-responsive GPBAR1 is expressed in cells of the intestine (predominantly ileum and colon), liver, brown adipose tissue, skeletal muscle, certain brain areas, pancreatic islets, and in immune cells such as macrophages (40–43). Following the binding of bile acids, GPBAR1 couples to Gαs, which activates adenylyl cyclase leading to increased intracellular cAMP levels, and the activation of cAMP-dependent effector proteins/kinases (41, 42, 44).

## 2 Effects of SCFA signaling on glucose homeostasis

SCFAs, including acetate, propionate, and butyrate, are small-molecule metabolites generated by gut microbiota through anaerobic fermentation of non-digestible carbohydrates. SCFAs are known to contribute to many metabolic disorders including obesity (45) and diabetes (46), suggesting that SCFAs affect multiple pathways involved in glucose/lipid metabolism and inflammation. As already mentioned above, SCFAs act on two major GPCRs, FFAR2 and FFAR3. Based on the expression profiles of FFAR2 and FFAR3, we focus on the signaling effects of SCFAs on glucose metabolism in different metabolically important tissues, including gut, pancreas, and adipose tissue.

### 2.1 SCFA effects in the gut

In the gut, where SCFA concentrations can reach 100 mM (47), SCFAs stimulate the release of anorectic hormones such as GLP-1 and peptide YY (PYY), in both mouse and human (48–50). GLP-1 is a well-known incretin hormone, released by enteroendocrine L-cells of the gut, and functions to lower blood glucose by directly stimulating insulin secretion from beta cells and inhibiting glucagon secretion from alpha cells of the pancreas. Accordingly, FFAR2 and FFAR3 are highly expressed in L-cells localized in the terminal ileum and colon (51). However, the co-expression of both GPCRs and their overlapping potency in sensing SCFAs has made it difficult to distinguish which of the two receptors is primarily responsible for regulating the secretion of gut hormones. However, the generation of animal knock-out (KO) models as well as the development of selective FFAR2 and FFAR3 agonists and antagonists has led to novel insights in this field.

In primary colonic cultures from FFAR2-KO mice, induction of GLP-1 release by SCFAs was severely impaired while the reduction was less pronounced in the absence of FFAR3 (52). Orthosteric FFAR2 agonists induced GLP-1 secretion in murine STC-1 enteroendocrine cells (53), whilst an allosteric FFAR3 agonist produced a modest stimulation of GLP-1 release from murine colonic crypt cultures (51). Given the signaling bias and low selectivity of these compounds, Bolognini et al. (54) constructed a hFFAR2-DREADD knock-in mouse by replacing mouse FFAR2 with a Designer Receptor Exclusively Activated by Designer Drugs (DREADD) derived from human FFAR2. The hFFAR2-DREADD is unresponsive to endogenous SCFAs but displays near equivalent signaling to wildtype (WT) hFFAR2 after activation by inert synthetic ligands, such as sorbic acid and 4-methoxy-3-methyl-benzoic acid (MOMBA) (55, 56). In agreement with related studies, activation of the FFAR2-DREADD induced GLP-1 release *in vitro* in primary colonic crypts from intact colon, and *in vivo* via intra-colonic administration of sorbic acid (54). Moreover, hFFAR2-DREADD was responsive to selective FFAR2 antagonists, and these antagonists efficiently blocked incretin production by sorbic acid and MOMBA (54). Taken together, these data suggest that FFAR2 but not FFAR3, mediates the stimulatory effects of SCFAs on GLP-1 release (54).

Moreover, mice with global knockout of FFAR2 showed reductions in colonic GLP-1 content and oral glucose stimulated insulin secretion, with no such metabolic defects observed in FFAR3-KO mice (52). This suggests that the observed impairment in glucose tolerance in FFAR2-KO mice may be partly due to decreased GLP-1 levels and impaired GLP-1-stimulated insulin secretion (52). Interestingly, in high fat diet (HFD)-fed mice treated with antibiotics to deplete gut microbiota, acetate-induced improvement in metabolic phenotypes were reversed upon FFAR2-deletion (57). This underscores the critical role of the gut microbiota, and their SCFAs in driving FFAR2’s effect on glucose and insulin tolerance. It is important to note that FFAR2 is not only expressed in colonic cells but also in other cell types such as pancreatic islet cells and adipocytes, as discussed below. Mechanistically, binding of SCFAs to FFAR2 leads to Gαq/11-dependent increases in intracellular calcium, thus triggering GLP-1 release in enteroendocrine cells (52). Additionally, FFAR2 internalization in endosomes activates the Gαi/p38 signaling pathway, which is also essential for propionate-induced GLP-1 release in colonic crypts and enteroendocrine cells (34). Regarding the metabolic role of intestinal FFAR3, Kristen and colleagues found no changes in circulating GLP-1 in mice with intestine-specific deletion of FFAR3 although these mice trended towards lower fasting glucose and insulin levels when maintained on a calorie-rich diet (58). The observed improvement in glucose homeostasis in the mice with intestine-specific deletion of FFAR3 may be indirectly mediated by reduced fat mass (58). Given the accumulating evidence that prebiotic diets enriched with fermentable dietary fibers can improve glucose homeostasis (59, 60) and the proven efficacy of GLP-1 receptor agonists in treating glucose impairment and obesity, targeting intestinal FFAR2 with specific drugs holds therapeutic potential for improving systemic glucose homeostasis.

### 2.2 SCFAs effects on pancreatic islets

Both FFAR2 and FFAR3 are expressed in islet beta cells in both rodents and humans (61), suggesting a role of SCFAs in regulating insulin secretion. Deletion of FFAR2 and/or FFAR3 do not affect glucose homeostasis in mice fed normal chow (57, 61–63). Studies regarding the role of FFAR2 in HFD-induced impairments in glucose homeostasis have yielded conflicting results, probably due to FFAR2 effects on various metabolic tissues.

Mice with global deletion of FFAR2 exhibited fasting hyperglycemia and glucose intolerance after HFD feeding, resulting from a defect in beta cell function and mass (63). The role of FFAR2 in glucose-stimulated insulin secretion (GSIS) was also corroborated by *ex vivo* and *in vitro* studies where the ability of a FFAR2 agonist to induce GSIS was abrogated in FFAR2-KO islets and in MIN6 cells treated with siRNA for FFAR2 or Gαq/11 (63). Using dynamic perifusion of mouse and human islets, acetate (1 mM) and propionate (1 mM) potentiated GSIS in a FFAR2-dependent manner, and this stimulatory effect was dependent on Gαq/11-induced calcium elevations and activation of PLC (64). Moreover, Gαq-biased FFAR2 agonists (SCA14 and SCA15) potentiated GSIS in islets from WT mice, but not in FFAR2-KO islets (65). These studies indicate that SCFAs stimulate GSIS through FFAR2-Gαq activation. On the other hand, other studies reported that activation of FFAR2 and/or FFAR3 inhibits insulin secretion through Gαi signaling. In beta cell lines and mouse islets, addition of acetate (0.1 to 1 mM) caused a dose-dependent decrease in GLP-1-stimulated insulin release or GSIS, which was blocked by either a Gαi inhibitor, pertussis toxin (PTX), or by reduced expression of FFAR2 and FFAR3 (61). Also, an FFAR3 agonist, 1-methylcyclopropane (MCPC) inhibited GSIS in WT islets, while FFAR3-KO islets showed increased GSIS compared to WT islets (66). Additionally, Gαi-biased FFAR2 agonists (4-CMTB and TUG-1375) inhibited GSIS in both mouse islets (65) and human pseudoislets (67). In agreement with the inhibitory role of FFAR2 and FFAR3 on insulin secretion, double-KO of FFAR2 and FFAR3 either globally or selectively in pancreatic beta cells strongly potentiated GSIS, resulting in improved glucose tolerance in HFD-fed mice (61). Similarly, compared to islets from WT mice, islets from FFAR3-KO mice secreted higher levels of insulin which correlated with improved glucose tolerance in the FFAR3-KO mice, whereas the opposite phenotypes were observed in FFAR3-overexpressing mice (68). The dual coupling of FFAR2 to Gαq and Gαi may explain the variable outcomes of studies of the effects of SCFAs on insulin secretion *in vitro* and *in vivo*. It is likely that Gαq-biased FFAR2 agonists, in combination with FFAR3 antagonists, could prove beneficial to enhance insulin secretion and improve glucose homeostasis. However, in this context, the paracrine effects of other islet cells including glucagon-secreting alpha cells and somatostatin-secreting delta cells on beta cell activity also need to be considered. Single-cell RNA sequencing data from human and mouse islets (69, 70) indicate that FFAR3 is highly expressed in alpha cells whereas FFAR2 is highly expressed in both alpha and delta cells. It has been shown that glucagon serves as a stimulator of insulin secretion through beta cell Gαs-coupled GLP-1 and glucagon receptors when glucose levels are high (71–73), while somatostatin inhibits insulin secretion through Gαi-coupled somatostatin receptor in beta cells (74, 75). Thus, the contribution of SCFAs on insulin release and glucose homeostasis in general from these non-beta cells deserves further study.

### 2.3 SCFA effects on adipose tissue function

Adipocytes play a crucial role in regulating glucose homeostasis through both endocrine mechanisms (via the release of adipokines, such as leptin and adiponectin, and non-esterified fatty acids) and non-endocrine mechanisms (fat mass changes through adipocyte metabolism) (76). In mice, white adipose tissues (WATs) mainly express FFAR2 (27, 57, 77). *In vitro* studies have shown that exogenous SCFAs inhibit lipolysis via activation of FFAR2 (78) in a PTX-sensitive fashion (54), indicating that FFAR2-mediated regulation of lipolysis is transduced by Gi signaling. However, the effects of FFAR2 on adipogenesis and glucose metabolism observed in FFAR2 global KO mice have produced conflicting results. On one hand, Bjursell et al. reported that FFAR2-KO mice (genetic background: C57BL/6JOlaHsd) fed a HFD showed reduced adiposity and increased lean mass, resulting in improved glucose control (62). On the other hand, Kimura et al. reported that HFD-fed FFAR2-KO mice (genetic background: 129/SvEv) displayed increased adiposity and impaired glucose homeostasis characterized by insulin resistance and glucose intolerance (57). Interestingly, transgenic mouse model with FFAR2 overexpression in adipocytes was resistant to HFD-induced body weight gain and exhibited attenuated insulin resistance (57). The discrepancies between the studies by Bjursell et al. and that of Kimura et al. may be due to variations in genetic background and the differing culture environments (57). Additionally, whole body FFAR2-KO may impact the compensatory expression of FFAR3 (62), which could contribute to the observed discrepancies. In terms of the underlying mechanism, Kimura et al. demonstrated that FFAR2 signaling is mediated by Gαi, and the inhibition of Akt phosphorylation by G(i/o)βγ suppressed insulin-mediated fat accumulation, leading to improved insulin sensitivity and energy utilization in other tissues such as liver and muscle, which ultimately led to improvement in systemic glucose homeostasis (57). Regarding FFAR3, studies with cultured adipocytes showed that SCFAs induce the expression of leptin following overexpression of FFAR3; this effect was suppressed after knockdown of FFAR3 expression (79). However, it still remains unclear whether FFAR3 is actually expressed in WAT at significant levels (27, 57, 77). So far, the adipocyte-specific effect of FFAR2 on glucose homeostasis and metabolic disorders is inconsistent and inconclusive. Considering the importance of FFAR2 and FFAR3 as crucial links between gut microbiota and systemic glucose homeostasis, it is important for future studies to clarify these reported discrepancies by examining conditional KO models of FFAR2 and FFAR3, such as adipocyte-specific KO.

## 3 Effect of dietary unsaturated fatty acids on glucose homeostasis

The following section on unsaturated fatty acids will focus on *cis*-configurated long-chain mono- and poly-unsaturated fatty acids that contain at least 12 carbons and are mostly found in plant-based vegetable oils, nuts, and seeds.

In mouse MIN6 beta cells, an array of unsaturated fatty acids including oleic, linoleic, eicosapentaenoic acid, and docosahexaenoic acid induced insulin secretion in a dose-dependent manner under hyperglycemic conditions (80). This effect was FFAR1-dependent, since siRNA knockdown of FFAR1 expression significantly reduced insulin secretion by linoleic acid, the most potent fatty acid agonist of FFAR1 in mice (80). In agreement with this observation, Schnell and colleagues found that long-chain mono- and polyunsaturated fatty acids increased intracellular Ca^2+^ levels and insulin release in primary mouse beta-cells and INS-1 cells; this effect was abrogated in INS-1 cells upon siRNA-mediated downregulation of FFAR1 (81). In a related study conducted with rat pancreatic beta-cells, oleic acid (1-10 μM) dose-dependently increased intracellular Ca^2+^ levels at increasing concentrations of glucose (82). Moreover, when rat beta-cells where transfected with FFAR1 siRNA, the oleic acid-induced increase in intracellular Ca^2+^ was impeded due to decreased PLC activity, resulting in impaired insulin release (82). Another study also reported that in isolated rat islets, linoleic acid dose-dependently stimulated glucagon release at both low (3 mM) and high (15 mM) glucose levels, although higher glucose concentrations expectedly decreased basal glucagon levels (83). However, inhibition of FFAR1 abrogated the effect of linoleic acid on glucagon release, along with decreased intracellular Ca^2+^ levels stemming from impaired PLC activity (83). Similarly, Flodgren and colleagues showed that linoleic acid dose-dependently increase glucagon secretion in isolated mouse islets, an effect which was annulled by antisense treatment against FFAR1 (84). The tendency of activated FFAR1 signaling to increase the release of both insulin and glucagon under hyperglycemic conditions appears surprising, given the fact that these two hormones are considered functional antagonists. However, recent studies using different experimental models have shown that glucagon release from alpha cells can promote insulin release via paracrine signaling under hyperglycemic conditions (85–88). FFAR1 is also expressed by incretin-releasing enteroendocrine cells (39). Following a HFD challenge, mice with global deletion of FFAR1 showed decreased plasma levels of GLP-1 and GIP, which led to a moderate decrease in plasma insulin and a concomitant increase in plasma glucose levels (39). These data suggest that insulin release following fatty acid activation of FFAR1 can occur directly or indirectly through action of incretins.

As already mentioned, FFAR4 is another GPCR activated by long chain fatty acids that contributes to the regulation of glucose homeostasis (35). Interestingly, morbidly obese subjects have reduced FFAR4 expression levels in visceral adipose tissue and in peripheral blood mononuclear cells (89). Another study reported that the anti-inflammatory effects of oleic acid and docosahexaenoic acid in human visceral adipocytes were mediated by FFAR4 (90), which has an indirect effect on insulin signaling and glucose homeostasis. In humans, a non-synonymous mutation of FFAR4 associated with obesity whereas global knockout of FFAR4 in HFD mice led to weight gain, glucose intolerance, fatty liver, and insulin resistance, suggesting that intact FFAR4 signaling is critical for maintaining systemic glucose homeostasis (91). In murine RAW264.7 and mouse primary intraperitoneal macrophages, docosahexaenoic acid and eicosapentaenoic acid repressed inflammation, and also reduced immune infiltration in adipose tissue of mice, along with improved insulin sensitivity, in a FFAR4-dependent manner (92). In line with this, when FFAR4-KO mice and WT controls were maintained on HFD supplemented with the selective FFAR4 agonist, Compound A, there was significant improvement in glucose tolerance and insulin sensitivity in the WT mice, but not in the FFAR4-KO mice (93). In mice, delivery of α-linoleic acid directly into the stomach increased plasma levels of GLP-1 and insulin in a FFAR4-dependent manner (94). Complementary experiments in STC-1 cells revealed that knockdown of FFAR4 but not FFAR1 reduced α-linoleic acid-mediated GLP-1 secretion (94). In isolated mouse pancreatic islets and clonal pancreatic BRIN-BD11 cells, α-linoleic acid, eicosapentaenoic acid, and docosahexaenoic acid mimicked the actions of a FFAR4-specific agonist, GW-9508, in stimulating insulin release, an effect that was accompanied by significant elevations in intracellular Ca^2+^ and cAMP (95). Accordingly, treatment (i.p.) of mice with α-linoleic acid augmented insulin secretion, leading to improved glucose tolerance (95).

The effect of FFAR4 activation by unsaturated fatty acids on glucagon release has also been explored in a limited number of studies. In islets isolated from whole-body FFAR4-KO mice, docosahexaenoic acid-induced glucagon secretion was significantly reduced, when compared to control mice (96). FFAR4 is also well expressed in somatostatin-producing delta cells in pancreatic islets (69, 97). In agreement with this observation, glucose-stimulated somatostatin secretion was significantly decreased by omega-3 long-chain PUFAs in pancreatic islets from WT mice (98) but not in islets from FFAR4-KO mice (97). However, the physiological relevance of this effect remains to be established.

Taken together, available evidence shows that unsaturated fatty acids, and especially omega-3 PUFAs influence glucose homeostasis by serving as potent ligands for FFAR1 and FFAR4 expressed by pancreatic islets and enteroendocrine cells.

## 4 Effects of dietary saturated fatty acids on glucose homeostasis

Saturated fatty acids (SFAs) lack double bonds in their hydrocarbon chain. Studies of the effect of SFAs on glucose homeostasis have focused on specific fatty acids such as palmitate. Palmitate is a highly abundant dietary SFA and known as very potent FFAR1 agonist (80). In humans, the intake of dietary fat rich in the palmitic acid leads to adverse effects on postprandial glycemic control, due to reduced β-cell function and decreased insulin sensitivity (99). A related study showed that 7-day exposure of human islets to 0.5 mM of palmitate impaired GSIS, most likely due to GLP-1 receptor-dependent upregulation of inflammatory markers such as *SOCS2, IL-1B*, and *TNFα* (100). This effect of palmitate on human islets was recapitulated in mouse MIN6 pseudoislets, but was abrogated following siRNA-mediated knockdown of *Socs2* (100). This observation agrees with the well-described pro-inflammatory effects of palmitate and other SFAs in human and murine cell types (101, 102) as well as the anti-inflammatory effects of GLP-1 receptor signaling (103, 104). In isolated mouse islets, palmitate induced glucagon secretion in a FFAR4-dependent manner, although the absence of FFAR4 had no effect on GSIS (96). Similarly, palmitate has been shown to stimulate glucagon secretion in WT mouse islets through enhanced Ca^2+^ entry into alpha cells to promote alpha-cell exocytosis (105). Clearly, additional studies are needed to explore the mechanisms underlying the effects of different SFAs on systemic glucose metabolism.

## 5 Effects of bile acids on glucose homeostasis

In humans that have undergone bariatric surgery to correct obesity, there exists a positive correlation between improved glucose metabolism and increased levels of circulating bile acids (106–108). It is now established that modulation of bile acid composition affects systemic glucose metabolism through alterations in intestinal lipid absorption or via activation of receptor-mediated signaling through farnesoid X-receptor (FXR) and GPBAR1 (109, 110). As a GPCR, GPBAR1, following the binding of bile acids, couples to Gαs (111). GPBAR1 shows highest affinity for lithocholic acid (EC_50_ ~0.53 μM), followed by conjugated and unconjugated forms of deoxycholic acid, chenodeoxycholic acid, and cholic acid (EC_50_ ~1.0, 4.4 7.7 μM, respectively) (109, 110, 112). Interestingly, mice with elevated levels of lithocholic and chenodeoxycholic acids show improved systemic glucose tolerance (113, 114), possibly through increased secretion of GLP-1 (115). This finding agrees with reports of relatively high expression of GPBAR1 in small intestine, colon, and enteroendocrine L-cells (116–118) (**Figure 1**), and the observation that treatment of STC-1 cells with GPBAR1 agonists enhances GLP-1 secretion (44). Additionally, GPBAR1 agonists improves whole-body glucose tolerance in mice, via increased secretion of GLP-1 and insulin (119). Hyocholic acid is a major primary bile acid in pigs, a specie noted for great resistance against type-2 diabetes (120). Based on this rationale, Zheng and colleagues investigated the anti-diabetic effect of hyocholic acid by demonstrating that the levels of hyocholic acid and blood glucose levels in humans with type-2 diabetes show an inverse correlation (120, 121). Mechanistically, hyocholic acid enhanced GLP-1 secretion by enteroendocrine cells through simultaneous activation of GPBAR1 and inhibition of FXR, leading to improved glucose tolerance. Moreover, hyocholic acid showed a stronger potency at stimulating cAMP production in comparison to the endogenous GPBAR1 agonist, lithocholic acid (121). In isolated mouse pancreatic islets, taurine-conjugated ursodeoxycholic acid (TUDCA) enhanced GSIS through the cAMP/PKA pathway (122). Similarly, in both human and mouse islets, as well as in MIN6 beta cells, the activation of GPBAR1 by lithocholic acid increased intracellular cAMP and Ca^2+^ levels and increased insulin secretion (123). In agreement with this finding, lithocholic acid and synthetic GPBAR1 agonists improved glucose tolerance in mice through increases in GLP-1 and insulin release (124), as well as increased L-cell differentiation and abundance (125). Moreover, the metabolic outcome of GPBAR1 expression/activation has been investigated in other important metabolically relevant tissues such as the skeletal muscle and brain. In the postprandial state, most circulating glucose is disposed by skeletal muscle, indicative of the key role of skeletal muscle in maintaining euglycemia (126–128). In mice, GPBAR1 activation enhanced insulin-stimulated glucose uptake by skeletal muscle (124), and skeletal muscle-specific overexpression of GPBAR1 improved glucose tolerance (129). Based on reports that bile acids reach the brain (130) and GPBAR1 is expressed in the central nervous system (40, 131), Perion and colleagues investigated the central effects of bile acids. They found that bile acids exert an anorexigenic effect by activating hypothalamic GPBAR1; however, this study did not measure the effects on systemic glucose homeostasis (132). Another study showed that reduced hypothalamic bile acid content in diet-induced obese mice can be counteracted by bile acid supplementation or by central administration of the selective TGR5 agonist, (2-chlorophenyl)-N-(4-chlorophenyl)-N,5-dimethyl-4-isoxazolecarboxamide, leading to decreased body weight and fat mass, via GPBAR1-mediated activation of the sympathetic nervous system (133). Although reduced adiposity is known to correlate with improved glucose tolerance, the authors did not assess effects on glucose metabolism. However, oral treatment of *ob/ob* mice with taurocholic acid resulted in improved glucose tolerance via activation of a gut-brain pathway, since the improved glucose tolerance depended on gut-derived FGF15 and intact melanocortin signaling (45).

Taken together, *in vivo* studies in humans and mice, as well as *in vitro* experiments with isolated islets and cultured cells demonstrate that specific bile acids show strong effects on glucose homeostasis through activation of GPBAR1, resulting in multiple beneficial metabolic effects including increased insulin secretion. These findings strongly suggest that targeting the bile acid-GPBAR1 axis may prove useful for the development of novel antidiabetic drugs.

## 6 Conclusion

Maintaining systemic glucose levels within a physiological range during fasting and feeding relies on the coordinated regulation of endogenously secreted insulin and glucagon. GPCRs that are responsive to specific bio-metabolites play a central role in regulating the release of these two important hormones. For example, SCFAs act on FFAR2 and FFAR3, bile acids on GPBAR1, and saturated and unsaturated fatty acids on FFAR1 and FFAR4, respectively (**Figure 1**). Most studies reported that SCFAs, bile acids, and unsaturated fatty acids have positive effects on glucose homeostasis, due in part by promoting incretin and insulin release, resulting in reduced glucose excursions in the post-prandial state. However, long chain SFAs seem to have adverse effects on systemic glucose homeostasis due to their pro-inflammatory effects and limited efficacy in inducing GPCR-mediated release of incretins and insulin. Among saturated fatty acids, the metabolic effect of palmitic acid has been studied in considerable detail, in contrast to other SFAs such as capric, lauric, myristic, and stearic acids. It would thus be of interest to interrogate the potential metabolic roles and signaling properties of these latter SFAs. The data discussed in this brief review indicate that GPBAR1 and FFARs 1, 2, 3 and 4 all represent potential pharmacological targets for improving impaired glucose homeostasis in type-2 diabetes and related metabolic disorders. More studies are needed to explore whether simultaneous targeting of several of these receptors might provide even greater therapeutic benefits. Additionally, because these GPCRs are expressed in multiple cells and tissues, the tissue- and cell-type specific effects of targeting these receptors need to be explored in more detail by using conditional and inducible mouse knockout models. This is especially true for FFAR2, due to the discordant reports obtained from studies in global KO mice. Such studies will provide important new insights into the mechanisms through which the different receptors affect glucose and lipid metabolism, providing a rational basis for the design of novel drugs aimed at treating various important metabolic diseases.

## Author contributions

A-BO and LL conceptualized and wrote the manuscript. A-BO is responsible for the final content of the manuscript. All authors contributed to the article and approved the submitted version.
